# Acoustic Properties Predict Perception of Unfamiliar Dutch Vowels by Adult Australian English and Peruvian Spanish Listeners

**DOI:** 10.3389/fpsyg.2017.00052

**Published:** 2017-01-27

**Authors:** Samra Alispahic, Karen E. Mulak, Paola Escudero

**Affiliations:** ^1^MARCS Institute for Brain, Behaviour and Development, Western Sydney UniversitySydney, NSW, Australia; ^2^Australian Research Council Centre of Excellence for the Dynamics of Language, Western Sydney UniversitySydney, NSW, Australia

**Keywords:** non-native speech perception, acoustic similarity, vowel inventory, vowel discrimination, vowel perception

## Abstract

Research suggests that the size of the second language (L2) vowel inventory relative to the native (L1) inventory may affect the discrimination and acquisition of L2 vowels. Models of non-native and L2 vowel perception stipulate that naïve listeners' non-native and L2 perceptual patterns may be predicted by the relationship in vowel inventory size between the L1 and the L2. Specifically, having a smaller L1 vowel inventory than the L2 impedes L2 vowel perception, while having a larger one often facilitates it. However, the Second Language Linguistic Perception (L2LP) model specifies that it is the L1–L2 *acoustic* relationships that predict non-native and L2 vowel perception, regardless of L1 vowel inventory. To test the effects of vowel inventory size vs. acoustic properties on non-native vowel perception, we compared XAB discrimination and categorization of five Dutch vowel contrasts between monolinguals whose L1 contains more (Australian English) or fewer (Peruvian Spanish) vowels than Dutch. No effect of language background was found, suggesting that L1 inventory size alone did not account for performance. Instead, participants in both language groups were more accurate in discriminating contrasts that were predicted to be perceptually easy based on L1–L2 acoustic relationships, and were less accurate for contrasts likewise predicted to be difficult. Further, cross-language discriminant analyses predicted listeners' categorization patterns which in turn predicted listeners' discrimination difficulty. Our results show that listeners with larger vowel inventories appear to activate multiple native categories as reflected in lower accuracy scores for some Dutch vowels, while listeners with a smaller vowel inventory seem to have higher accuracy scores for those same vowels. In line with the L2LP model, these findings demonstrate that L1–L2 acoustic relationships better predict non-native and L2 perceptual performance and that inventory size alone is not a good predictor for cross-language perceptual difficulties.

## Introduction

In adulthood, perception of sound categories in a second language (L2) is broadly thought to occur through the lens of the native language (L1). That is, L2 sound categories are mapped to categories of the L1 (Best, 1995; Flege, 1995, 2003; Escudero, 2005, 2006, 2009; Best and Tyler, 2007). L2 perception difficulties are thus thought to arise from a lack of one-to-one mappings of categories between the L2 and the L1—for example, when two L2 sound categories map to a single L1 category, as in Japanese listeners' mapping of English /r/ and /l/ to the single Japanese category, /ɹ/. As difficulty in the perception of certain L2 sounds can extend to difficulties in recognizing words containing the same sounds, it is important to consider how and to what extent L1 and L2 sound inventories interact in L2 perception.

The relationship between the size of the L1 and L2 vowel inventory may predict non-native and L2 vowel perception (Fox et al., 1995; Lengeris, 2009; Bundgaard-Nielsen et al., 2011). In this view, having fewer L1 vowels than the target L2 will result in more perceptual difficulties, as more than one L2 vowel will be categorized to some L1 categories. That is, a consequence of a smaller vowel inventory is the fact that two vowels in a non-native category will be perceived as one single sound. By extension, having more L1 vowel categories than the L2 should facilitate L2 perception, since there are sufficient L1 categories for all L2 sounds to map to without the need for two L2 sounds to map to a single category. There is ample evidence demonstrating that L2 learners frequently struggle with sounds not present in their L1 (Fox et al., 1995; Flege et al., 1997; Escudero and Boersma, 2002; Morrison, 2003; Escudero, 2005). For instance, Mexican Spanish listeners, having a small five-vowel inventory, categorized Canadian English /i/ and /ɪ/ vowels to their single /i/ native category (Morrison, 2002). By the same token, individuals whose L1 vowel inventory contains more sound categories than the target language have been shown to outperform listeners with fewer first-language sounds. For example, native speakers of German and Norwegian—two languages that have a larger and more complex vowel system than English—identified English vowels more accurately than French and Spanish native speakers, whose L1 vowel inventories are smaller than that of English (Iverson and Evans, 2007, 2009). However, in this case, native speakers of all four languages relied on primary acoustic cues, such as F1/F2 formant frequencies, formant movement and duration in their perception of the English vowels, despite formant movement and duration not being present in Spanish and French, suggesting that in addition L1 vowel inventory size affecting perceptual accuracy, other acoustic-phonetic properties are also at play (Iverson and Evans, 2007, 2009). Together, these findings further suggest that while the scope of a learner's L1 vowel inventory may affect their L2 perceptual patterns, inventory size alone is not enough to accurately predict complexities of L2 perceptual patterns.

While several theories have proposed that L1–L2 relationships affect perception, they differ in some ways. The Speech Learning Model (SLM: Flege, 1995, 2003) proposes that non-native phonemes are perceived in accordance with learners' L1 acoustic properties. However, its focus lies predominantly on advanced learners' perception of individual phonemes, rather than naïve learners and vowel contrasts. The Perceptual Assimilation Model (PAM: Best, 1995), its extension to L2 learning (PAM-L2: Best and Tyler, 2007) and the Second Language Linguistic Perception model (L2LP: Escudero, 2005, 2006, 2009) focus on naïve listeners' perception of non-native and L2 contrasts, and propose that the features of listeners' native phonemes predict whether and to what extent contrasts will be discriminated and learned during L2 acquisition. However, PAM and PAM-L2 propose that it is the articulatory similarity/dissimilarity between L1–L2 sounds that influence and predict naïve listeners' non-native sound perception and later L2 development. The L2LP model (Escudero, 2005, 2006, 2009) is a computational model that takes into account listeners' learning trajectory from the initial state to ultimate attainment. It proposes that listeners will initially perceive non-native and L2 sounds in line with the acoustic features of their L1 sound system (Escudero and Chládková, 2010; Escudero et al., 2014). The model further specifies that apart from the number of vowels in a listener's L1 relative to the L2, detailed acoustic-phonetic comparisons between the L1 and L2 determine listeners' perceptual mapping and discrimination of non-native sounds.

L2LP posits that acoustic comparisons should ideally be quantitative measures of cross-linguistic similarity as this will allow for predictions of listeners' initial state of the overall L2 learning process, as this is the perceptual system that learners will initially use (Escudero, 2005). One method of quantifying cross-linguistic acoustic similarity is through linear discriminant analyses (LDA) models. In order to make initial L2 perceptual difficulty predictions, LDA models allow for cross-language similarity to be established independent of listeners' identification or discrimination performance (e.g., Strange et al., 2004, 2005; Gilichinskaya and Strange, 2010). However, some studies that have used LDA models have claimed that acoustic comparisons are not always good predictors of cross-language speech perception. For instance, in an examination of phonetic similarity between the first three formants of North German and American English vowels (Klecka, 1980), acoustic similarities between American English and North German vowels did not always predict perceptual similarity (Strange et al., 2005, 2004). In contrast, using the same discriminant analyses as Strange et al. (2004, 2005), a more recent study established that acoustic similarities were a good predictor of categorization patterns of American English vowels by Russian listeners (Gilichinskaya and Strange, 2010). Likewise, recent research has indeed shown that the L1/L2 acoustic relationship affects sound perception (e.g., Vasiliev, 2013; Elvin et al., 2014; Escudero et al., 2014). For example, despite the fact that Iberian Spanish listeners have a smaller vowel inventory in comparison to Australian English, they outperformed AusE listeners in their discrimination of six Brazilian Portuguese vowel contrasts (Elvin et al., 2014).

To first establish the effects of vowel inventory size vs. acoustic properties in non-native vowel perception, AusE listeners' XAB discrimination of five Dutch vowel contrasts (/a-ɑ/, /ɪ-i/, /y-ʏ/, /i-y/, and /ɪ-ʏ/) were compared to Peruvian Spanish (PS) listeners who took part in the same XAB task as reported in Escudero and Wanrooij (2010). As listeners' discrimination patterns should be predicted by their categorization patterns, listeners' categorization of the same target vowels was then compared to those of PS listeners reported in Escudero and Williams (2011). As shown in Table 1, AusE and PS vary not only in the number of phonemes present in each vowel inventory, but also in their F1, F2, and F3 acoustic properties. If vowel inventory size is indeed a reliable predictor of nonnative vowel perception, AusE listeners, whose vowel inventory is larger than that of PS, should outperform PS listeners in their discrimination of the five Dutch contrasts (/a-ɑ/, /ɪ-i/, /y-ʏ/, /i-y/, and /ɪ-ʏ/). However, the L2LP model states that acoustic-phonetic similarities between the native and target language predict perceptual mapping patterns^1^ and outlines different learning scenarios that predict discrimination difficulties prior to testing. For instance, a difficult scenario of L2 learning is the *New Scenario* whereby listeners perceive two target language sounds in line with a single native category (Escudero, 2005). Given that four of the Dutch vowels presented, namely /ɑ/, /ɪ/, /y/, and /ʏ/, are not part of the Spanish vowel inventory, it is expected that Spanish listeners will find contrasts containing these sounds (e.g., /ɑ-a/, /ɪ-i/, and /ʏ-y/) relatively difficult to discriminate, and are likely to categorize these across single native categories, namely /a/, /i/, and /e/, resulting in New Scenario. An easier pattern of discrimination occurs when listeners equate two L2 sounds with two L1 categories, referred to as *Similar Scenario* (Escudero, 2005). AusE listeners should find at least two Dutch contrasts less difficult than PS listeners, as AusE contains two /ɪ-i/ and two /ɐ, ɐː/ vowels. Additionally, as Dutch /y/ and /ʏ/ are not present in the AusE inventory it is further predicted that AusE will encounter *Subset Scenario* by equating each of these sounds to two or more native categories (Escudero and Boersma, 2002). This scenario often occurs for listeners with larger vowel inventories and difficulty is predicted to be higher than for New Scenario. That is, if perceptual overlap between the non-native and native categories occurs, then listeners are predicted to perceive a non-native contrast as the same multiple native categories. However, if no perceptual overlap occurs then the learning scenario should be easier than New Scenario to discern, but should not be easier to discern compared to Similar Scenario.

**Table 1:** **Male speakers' acoustic measures in Hertz of languages of the present study (AusE: Elvin et al., 2016; PS: Chládková et al., 2011; Dutch: Adank et al., 2004a)**.

**Language**	**Vowel**	**Measure**		
		**F1 (Hz)**	**F2 (Hz)**	**F3 (Hz)**
Australian English	/i:/	320	2339	2948
	/ɪ/	332	2336	2968
	/e/	467	2085	2799
	/æ/	695	1763	2669
	/ɐ:/	757	1349	2582
	/ɐ/	743	1386	2581
	/ɔ/	584	1040	2540
	/o:/	439	846	2575
	/ʊ/	378	948	2490
	/ʉ:/	341	1796	2427
	/ɜ/	468	1637	2581
	/ɪə/	329	2343	2980
	/e:/	452	2092	2792
	/ɑe/	660	1099	2557
	/æı/	745	1613	2617
	/oı/	480	956	2530
	/æɔ/	698	1844	2676
	/əʉ/	636	1442	2527
Peruvian Spanish	/a/	612	1356	2337
	/e/	455	1929	2532
	/i/	323	2186	2789
	/o/	483	942	2315
	/u/	371	824	2356
Dutch	/i/	278	2162	2665
	/ɪ/	361	1919	2536
	/a/	670	1425	2485
	/ɑ/	578	1172	2435
	/y/	259	1734	2205
	/ʏ/	366	1595	2345

If listeners' L1 vowel inventory size affects non-native discrimination difficulty, AusE listeners are predicted to outperform PS listeners overall in their discrimination of the Dutch vowel contrasts. This is in contrast to acoustic comparisons, where comparable perceptual difficulties across both listener groups would be expected. All of the aforementioned studies that used LDAs as a means of testing the predictive nature of listeners' L2 perception only tested listeners whose L1 vowel inventory is smaller than that of the L2. Thus, we further used LDA models to test whether acoustic similarities are predictive of categorization patterns by listeners with a smaller and larger vowel inventory compared to the target language (Strange et al., 2004, 2005; Gilichinskaya and Strange, 2010; Escudero and Vasiliev, 2011). As described in the Methods section, these analyses model AusE and PS listeners' likely classification patterns of Dutch vowels, and in turn predict their likely discrimination difficulties (Strange et al., 2004, 2005; Gilichinskaya and Strange, 2010; Escudero and Vasiliev, 2011). Table 2 presents the AusE and PS cross-language classification data percentages of the most frequent Dutch vowel classification to an AusE word and PS vowel.

**Table 2:** **Percentage of Dutch vowel token classification to an AusE word and PS vowel based on overall classification patterns of cross-language LDA**.

**Dutch vowel**	**AusE classifications**												**PS classifications**				
	**Dress**	**Fleece**	**Foot**	**Goose**	**Kit**	**Lot**	**Nurse**	**Palm**	**Square**	**Strut**	**Thought**	**Trap**	**/a/**	**/e/**	**/i/**	**/o/**	**/u/**
	**/ε/**	**/iː/**	**/ʊ/**	**/ʉː/**	**/ɪ/**	**/ɔ/**	**/ɜː/**	**/ɐː/**	**/eː/**	**/ɐ/**	**/oː/**	**/æ/**					
/a/						5	35	45		5		10	90	10			
/ɑ/	5		15			60				15		5	55			45	
/ɪ/				10	90									5	95		
/i/		25		10	65										100		
/ʏ/	5			75	20									70	30		
/y/				75	25										100		

Furthermore, the L2LP model posits that when distinguishing between L2 categories, listeners employ multiple sources of acoustic-phonetic information in their perception of phonological segments (Escudero, 2005). Previous research has indeed demonstrated that close attention is paid to the most salient acoustic cue of a particular sound (see Curtin et al., 2009; Mayr and Escudero, 2010; Escudero et al., 2014). For instance, Salento Italian listeners' perceptual patterns of standard Southern British English vowels were tested to establish their initial state in the acquisition of the Southern British English vowel system (Escudero et al., 2014). The results suggest that Southern British English vowels were initially mapped relative to the acoustic properties of the listeners' native vowel system. For example, the first two formants of Southern British English /ɪ/ and /ɔː/ fall between Salento Italian /i-e/ and /o-u/, respectively. However, Salento Italian listeners perceived these sounds as corresponding to their native /i/ and /o/ categories, displaying the use of single acoustic dimensions in their categorization. That is, F2 was the defining acoustic measure for English /ɪ/, and F3 for /ɔː/ (Escudero et al., 2014). Thus, to test classification power of each individual acoustic measure in our study, we conducted additional stepwise discriminant analyses in each language based on F1, F2, F3 as well as duration. Table 3 presents the AusE and PS cross-language classification data based on individual acoustic dimensions.

**Table 3:** **Percentage of Dutch vowel token classification to an AusE word and PS vowel based on classification patterns of individual dimension cross-language LDAs**.

**Measure**	**Dutch vowel**	**AusE classifications**												**PS classifications**				
		**Dress**	**Fleece**	**Foot**	**Goose**	**Kit**	**Lot**	**Nurse**	**Palm**	**Square**	**Strut**	**Thought**	**Trap**	**/a/**	**/e/**	**/i/**	**/o/**	**/u/**
		**/ε/**	**/iː/**	**/ʊ/**	**/ʉː/**	**/ɪ/**	**/ɔ/**	**/ɜː/**	**/ɐː/**	**/eː/**	**/ɐ/**	**/oː/**	**/æ/**					
F1 Bark and duration	/a/	5						5	60	10	5	10	5	90	5		5	
	/ɑ/	35		15			35				5		10	80	10		10	
	/ɪ/					100										45		55
	/i/				60	40										100		
	/ʏ/			5	10	85									10	45		45
	/y/				85	15										100		
F1, F2 Bark and duration	/a/						5	30	60		5			90	10			
	/ɑ/	5		15			60				20			55			45	
	/ɪ/				5	95									5	95		
	/i/		25		5	70										100		
	/ʏ/	5			80	15									65	30		5
	/y/				85	15										90		10
F1, F2, F3 Bark and duration	/a/						5	35	45		5		10	90	10			
	/ɑ/	5		15			60				15		5	55			45	
	/ɪ/				10	90									5	95		
	/i/		25		10	65										100		
	/ʏ/	5		15	60	20									65	30		5
	/y/			15	60	25										90		10

Based on the cross-validation classification sets in Tables 2, 3 our predicted perceptual patterns for each Dutch contrast by AusE and PS listeners are as follows:

- Dutch /ɪ*-i*/
  It is expected that both listener groups should face comparable difficulties in their discrimination of the Dutch /ɪ-i/ contrast. Listeners are predicted to predominantly perceive these two non-native vowels in line with a single native category, namely AusE /ɪ/ and PS /i/. Additionally, based on the stepwise classifications AusE /i/ exhibits F2 and F3 acoustic similarity to Dutch /i/ more than AusE /ɪ/. Therefore, AusE listeners are further predicted to exploit these differences and are expected to categorize Dutch /i/ as AusE /i/ some of the time, in addition to AusE /ɪ/. PS listeners are predicted to also exploit F2 and F3 by categorizing Dutch /ɪ/ as PS /e/ and /u/.
- *Dutch /ʏ-y*/
  Based on the overall LDA classifications, AusE listeners are expected to encounter New Scenario in their discrimination of the Dutch /ʏ-y/, as they are likely to perceive both vowels as AusE /ʉː/. However, based on the stepwise DAs, these vowels are acoustically similar to AusE /ʉ:/, /ɪ/ and /ʊ/ (F1), /ε/, /ʉ:/ and /ɪ/ (F2), and /ε/, /ɪ/, /ʊ/ and /ʉ:/ (F3). Thus, in line with these parameters, AusE listeners are predicted to encounter the Subset Scenario by categorizing Dutch /ʏ-y/ across multiple native categories, namely AusE /ε/, /ɪ/, /ʊ/, and /ʉ:/. PS listeners are predicted to not face difficulties in their discrimination of Dutch /ʏ-y/ and are expected to encounter Similar Scenario by perceiving both sounds in line with a distinct native phoneme namely PS /e/ and /i/.
- *Dutch* /*i*-*y*/
  In line with overall perceptual similarity, PS listeners are predicted to categorize Dutch /i-y/ as PS /i/. However, based on the stepwise classifications, Dutch /y/ also exhibits some F2 and F3 similarity to PS /u/. While minimal, these differences between backness and rounding are predicted to aid PS listeners when discerning these two sounds. Similarly, AusE listeners are predicted to exploit all three acoustic parameters in their perception of Dutch /i-y/. That is, based on height (F1), Dutch /i/ is acoustically most similar to AusE /ɪ/ and /ʉ:/, while Dutch /y/ is acoustically most similar to AusE /ʉ:/. However, based on the first two formants Dutch /i/ is closest to AusE /i/, while Dutch /y/ is most similar to AusE /ʉ:/. Therefore, AusE listeners are predicted to face Similar Scenario by categorizing Dutch /i-y/ as AusE /ɪ/ and /ʉ:/. This learning scenario is also predicted by the cross-linguistic DA classification patterns.
- Dutch /ʏ-ɪ/
  Based on overall LDA classifications, both listener groups are expected to encounter Similar Scenario when differentiating Dutch /ʏ-ɪ/, as these vowels are acoustically similar to distinct native phonemes. However, based on the stepwise DAs both Dutch /ʏ/ and /ɪ/ bear F1 similarity to AusE /ɪ/, F2 similarity to AusE /ʉ:/ and /ɪ/, and F3 similarity to AusE /ɪ/, /ʉ:/ and /ʊ/. However, based on classification percentages (e.g., F2: Dutch /ʏ/ → AusE /ʉ:/, 80% and /ɪ/ 15%, Dutch /ɪ/ → AusE /ɪ/, 95% and /ʉ:/, 5%), it is predicted that AusE listeners, facing Subset Scenario, should differentiate these two phonemes due to the due to the low acoustic overlap between the F2 and F3 cues. In contrast, PS listeners are predicted to exhibit lower discrimination accuracy compared to AusE listeners due to a higher acoustic overlap across all three acoustic dimensions and are further expected to classify both vowels as PS /i/, /e/, and /u/.
- Dutch /a-ɑ/
  In line with the overall DA and stepwise models, AusE listeners are predicted to find the Dutch /a-ɑ/ somewhat challenging to discern as these vowels were classified across two or more AusE vowel categories, while PS listeners are predicted to encounter New Scenario by predominantly mapping the sounds in line with PS /a/.

In sum, if predictions based on listeners' L1 vowel inventories size are borne out, AusE listeners, whose vowel inventory is larger than that of PS, are expected to have higher discrimination accuracy than PS listeners for all five Dutch contrasts. Cases of New Scenario are predicted for PS and that of Similar Scenario for AusE listeners. Alternatively and following L2LP's acoustic hypothesis, if acoustic differences between L1 and L2 influence non-native sound perception, both listener groups' discrimination difficulties should yield comparable results. That is, both listener groups are expected to face the New, Similar, and Subset Scenarios. To test these contrastive hypotheses, naïve AusE listeners' XAB discrimination and categorization of five Dutch vowel contrasts (/a-ɑ/, /ɪ-i/, /y-ʏ/, /i-y/, and /ɪ-ʏ/) was compared to those of naïve PS listeners reported in previous studies (Escudero and Wanrooij, 2010; Escudero and Williams, 2011).

## Methods

### Participants

Twenty-two monolingual AusE students aged 18–45 years (*M*_age_ = 24.1 years; 11 females) participated for course credit at Western Sydney University. Participants were born and raised in Greater Western Sydney, and reported no experience with Dutch or any hearing impairment.

Non-native vowel categorization data from the same AusE listeners were compared to non-native vowel categorization data from 40 PS monolinguals (20 females) from Lima, Peru reported in Escudero and Williams (2011). Participants ranged in age from 18 to 30 years^2^, and reported no knowledge of Dutch or hearing impairment. XAB discrimination data from our AusE listeners was then compared to discrimination data of 22 PS listeners reported in Escudero and Wanrooij (2010). Listeners were monolinguals aged 17–28 years (*M*_age_ = 20.95; 10 females) born and raised in Lima, Peru their entire life and reported no knowledge of Dutch.

Participant data collection for the present study was carried out in accordance with the Human Research Ethics Committee (HREC), Western Sydney University, approval number H9373.

### Stimuli and procedure

Both groups of participants first completed a two-alternative forced choice XAB discrimination task followed by a nonnative categorization task. The auditory stimuli for the XAB discrimination task were 20 naturally produced tokens of each of the five Dutch vowels /a/, /a/, /ɪ/, /i/, /y/, and /ʏ/, extracted from recordings produced by 20 native Standard Northern Dutch speakers (10 females) in monosyllabic utterances in a neutral non-word /sVs/ consonantal context embedded within a carrier sentence (Adank et al., 2004b). In the XAB task, listeners heard three sounds in a row and were then asked to indicate whether the first sound (X) sounded more like the second (A) or third (B) sound by clicking on one of two yellow squares (viz. “2” and “3”) presented on a computer screen. There was an inter-stimulus interval of 1.2 s, which was selected because it is long enough to trigger phonological activation (Werker and Logan, 1985; Van Hesse and Schouten, 1999; Escudero and Wanrooij, 2010), and an inter-trial interval of 0.5 s following the participant's selection. The experiment was conducted in Praat and consisted of five blocks (one for each contrast—/a-ɑ/, /ɪ-i/, /y-ʏ/, /i-ʏ/, and /ɪ-ʏ/) containing 80 trials each.

Stimuli for the non-native vowel categorization task were 20 naturally produced tokens of each of the 12 Dutch monophthongal vowels, /ɑ, a, e, ε, ɪ, i, ɔ, o, ø, u, ʏ, y/, extracted from the same speakers and context as in the XAB task. As the present study compares non-native discrimination and categorization, we report categorization results only for the same vowels presented in the XAB task. The task consisted of 240 randomized test trials and participants completed 12 practice trials prior to beginning. In each trial, PS listeners were asked to categorize one Dutch vowel token to one of the nine PS (/a, e, i, o, u, ei, eu, ue, ou/) and 12 AusE (/i:, ɪ, e, e:, ɑe, ɐ:, ɐ, ɔ, o:, ʊ, ʉ:, ɜ:/) vowels presented orthographically on the screen. According to the Orthographic Depth Hypothesis (ODH; Katz and Frost, 1992), Spanish has a very straightforward correspondence between phonemes and their graphemic representations (Escudero and Wanrooij, 2010). That is, each grapheme tends to represent one phoneme only. However, English is not orthographically transparent and vowels can't reliably be presented using orthography unless they are embedded in words. Therefore, AusE listeners were asked to categorize the vowel to native words that each contained an AusE vowel (*heed, hid, hood, who'd, hair, head, heard, hall, had, hut, hot, hard*). There was a between-trial interval of 1 s and listeners could take a short break after every 24 trials.

Stimuli for both tasks were presented through headphones at a comfortable hearing level. Testing of AusE participants took place in a quiet room at the MARCS Institute, Western Sydney University. PS participants were tested in a quiet room at the Pontificia Universidad Católica del Perú, in Lima (Escudero and Wanrooij, 2010; Escudero and Williams, 2011). Before starting each task, listeners completed a practice session to familiarize themselves with the testing procedure. Each listener took ~1 h to complete both tasks.

### Linear discriminant analyses

We implemented a vowel-intrinsic normalization procedure where the first three formant values for each language were converted from the Hertz to the Bark scale using Traunmüller (1990) critical band rate Equation (1) (see Syrdal and Gopal, 1986). This procedure is typically used for modeling human vowel perception, compared to a vowel-extrinsic procedure, which is traditionally used for automatic speech recognition purposes (Gerstman, 1968; Lobanov, 1971; Nordström, 1976; Nearey, 1978; Adank et al., 2004a).

(1) $$\begin{array}{r} F\frac{B}{i}=26.81\times \left(\frac{F_{i}}{1960+F_{i}}\right)-0.53. \end{array}$$

Two separate LDA models were first trained using the cross-validation method reported in Strange et al. (2004, 2005). Each LDA included F1/F2/F3 bark values and vocalic duration as an additional parameter as well as the six (/a/, /ɑ/, /ɪ/, /i/, /y/, /ʏ/) target L2 Dutch vowels (AusE: Elvin et al., 2016; PS: Chládková et al., 2011; Dutch: Adank et al., 2004b).

### Statistical analysis

A mixed-effects logistic model examining listeners' correct and incorrect responses was used to establish any effects of vowel inventory size and acoustic properties on L2 perception of all non-native Dutch vowel contrasts presented in the XAB task. In particular, we analyzed participants' correct responses, with participant, speaker, and XAB trial as random effects, and vowel contrast and language background as fixed effects. As a means of establishing discrimination ranking of contrasts both within and between participant groups, we then conducted further *post-hoc* pairwise comparisons. The statistical model was chosen as it is appropriate for evaluating data of categorical nature (see: Baayen et al., 2008; Jaeger, 2008; Arnon, 2010).

## Results

### Cross-language discriminant analyses

LDA models yielded 84.2% overall correct classification for AusE and 96.7% for PS. Percentages of the most frequent Dutch vowel classification to an AusE word and PS vowel are presented in Table 2. To inform the contribution of duration, we additionally ran two LDA models that did not include duration as a factor. While the classification parameters remained the same the models yielded slightly lower correct classification percentages when duration was removed; 72.2% overall correct classification for AusE and 94.05% for PS.

Two additional (one per language group) stepwise classification models were then trained and tested. Each step in the model contained the same acoustic parameters, vocalic duration as well as the same six target L2 Dutch vowels as the LDA models. The AusE stepwise DA yielded 33.9% for F1 and duration, 71.3% for F1, F2, and duration, as well as 73.3% for F1, F2, F3, and duration correct classification. Whereas, the PS model yielded 55.1 for F1, 87.7% for F1, F2, and duration, in addition to 90.1% for F1, F2, and F3 correct classification.

### Non-native vowel categorization

Table 4 presents the percentage of times (>5%) a Dutch vowel token was classified to an AusE and PS vowel. Instances of the New Scenario were observed for both groups, whereby two non-native Dutch sounds were mapped to a single native category: PS participants categorized both Dutch /i/ (94%) and /y/ (59%) to the single PS /i/ and both Dutch /ʏ/ (53%) and /ɪ/ (49%) sounds to PS /e/. AusE listeners mainly classified Dutch /ɪ/ (40%) and /i/ (48%) to AusE /ɪ/, while PS participants classified Dutch /i/ as their native PS /i/ (94%). PS participants classified Dutch /ɪ/ across two native categories, namely /i/ (39%) and /e/ (49%). AusE listeners mostly mapped Dutch /a/ to an acoustically similar AusE counterpart, /ɐː/ (47%), while Dutch /ɑ/ was mapped most frequently to AusE /ɔ/ (40%). PS listeners categorized Dutch /ɑ/ as PS /a/ (59%) and /o/ (33%), while Dutch /a/ was mapped to PS /a/ (96%). Furthermore, instances of the Subset Scenario, which involves non-native vowels being categorized as more than two native categories, was observed for AusE listeners e.g., /ʏ/ → /ε/ (19%), /ʊ/ (19%), /ʉː/ (14%) and /y/ → /ʉː/ (28%), /ʊ/ (17%), /ɪ/ (17%). PS listeners categorized these sounds mainly across two acoustically distinct native categories, PS /e/ (53%) and /i/ (59%), encountering Similar Scenario.

**Table 4:** **Categorization percentages of non-native Dutch vowels to AusE words by AusE listeners tested in the present study and to PS vowels by PS listeners as reported in Escudero and Williams (2011)**.

**Dutch stimuli**	**AusE responses**												**PS responses**								
	**Heed**	**Hid**	**Head**	**Heard**	**Hair**	**Had**	**Hard**	**Hall**	**Hut**	**Hot**	**Hood**	**Who'd**	**/i/**	**/e/**	**/ei/**	**/eu/**	**/a/**	**/o/**	**/ou/**	**/u/**	**/ue/**
	**/iː/**	**/ɪ/**	**/ε/**	**/ɜː/**	**/eː/**	**/æ/**	**/ɐː/**	**/oː/**	**/ɐ/**	**/ɔ/**	**/ʊ/**	**/ʉː/**									
/a/				6	10	15	47	6									96				
/ɑ/						9	13	7	13	40							59	33			
/ɪ/	8	40	20								6	6	39	49						7	
/i/	28	48	8										94								
/ʏ/		13	19	10	6						19	14	10	53						25	
/y/		17									27	28	59							32	

### XAB discrimination task

**Figure 1:**
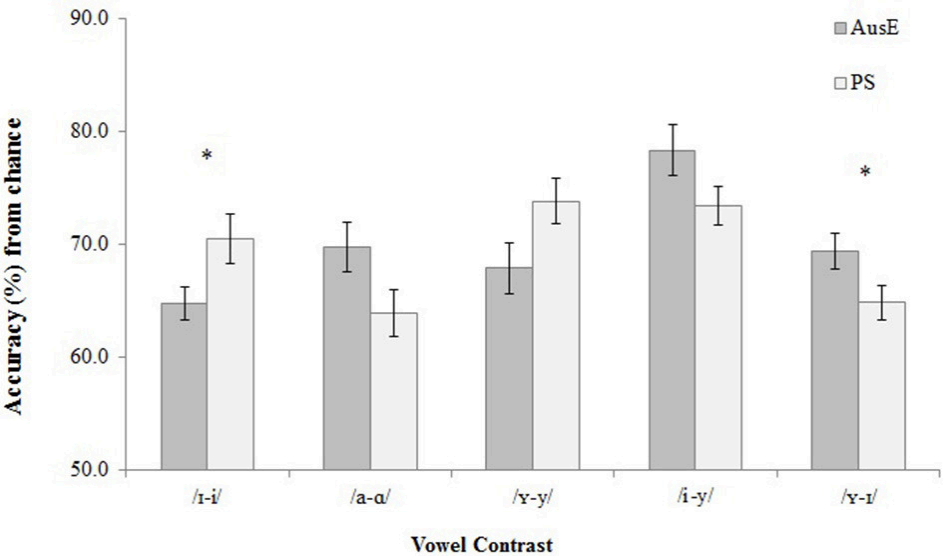
**Accuracy in discrimination of all non-native Dutch vowel contrasts by 22 native AusE and 22 native PS participants**. Standard error bars were treated as Independent Variables. Interaction of vowel contrast and language background is labeled (^*^).

To determine whether discrimination differed between participants whose native language had more (AusE) or fewer (PS) vowels compared to Dutch, we compared performance between AusE and PS listeners. A mixed-effects binary logistic model analyzing participants' correct responses, with participant, speaker, and XAB trial as random effects, and vowel contrast and language background as fixed effects revealed a main effect of contrast [$\chi_{\left(4,\;\text{N}\;=\;17,600\right)}^{2}$ = 38.7, *p* = < 0.001]. While there was no main effect of language background [$\chi_{\left(1,\;\text{N}\;=\;17,600\right)}^{2}=$ 0.112, *p* = 0.738], there was an interaction of vowel contrast and language background [$\chi_{\left(4,\;\text{N}\;=\;17,600\right)}^{2}$ = 16.5, *p* = 0.002]. Fishers' LSD-corrected *post-hoc* pairwise comparisons revealed that PS listeners had marginally more correct responses than AusE listeners for /ɪ-i/ (*p* = 0.053, 95% CI [−0.52, −0.003]), whereas AusE participants were marginally more correct for /ʏ-ɪ/ than PS listeners (*p* = 0.086 [−0.44, 0.03]). Figure 1 presents listeners' discrimination accuracy of the five non-native Dutch vowel contrasts.

Fisher's LSD-corrected *post-hoc* pairwise comparisons revealed that AusE participants had more correct responses for /i-y/ than /ɪ-i/, (*p* < 0.001, 95% CI [−0.09, −0.18]), /a-ɑ/, (*p* = 0.002, [−0.14, 0.03]), /ʏ-y/, (*p* < 0.001, [−0.16, −0.05]), and /ʏ-ɪ/ (*p* < 0.001, [−0.12, −0.06]); for /ʏ-ɪ/ than /ɪ-i/ (*p* = 0.043, [−0.09, 0.00]); and for /a-ɑ/ than /ɪ-i/ (*p* = 0.046, [−0.10, 0.00]). PS participants had more correct responses for /ʏ-y/ than /a-ɑ/ (*p* = 0.002, 95% CI [−0.16, CI [−0.16, −0.04]) and /ɪ-ʏ/ (*p* = 0.002, [−0.15, −0.03]); for /i-y/ than /a-ɑ/ (*p* = 0.003, [−0.16, −0.03]), and /ʏ-ɪ/ (*p* < 0.001, [−0.13, −0.04]); and for /ɪ-i/ than /ʏ-ɪ/ (*p* = 0.043, [−0.11, 0.00]), and trended toward more correct responses for /ɪ-i/ than /a-ɑ/ (*p* = 0.081, [−0.11, 0.00]). Table 5 presents listeners' discrimination ranking from most to least difficult contrast, along with mean accuracy percentages.

**Table 5:** **Difficulty ranking, mean accuracy percentages and standard error (SE) for XAB Discrimination task ranging from most (1) to least difficult Dutch vowel contrast by 22 native AusE and 22 native PS listeners**.

**L1 listener group**	**Dutch vowel contrast (% accuracy)**					
**Difficulty ranking**	**AusE**			**PS**		
1	/ɪ-i/	(64.8%)	SE 1.4	/a-ɑ/	(63.9%)	SE 2.1
				/ʏ-ɪ/	(64.9%)	SE 1.5
2	/ʏ-y/	(67.9%)	SE 2.3	/ɪ-i/	(70.5%)	SE 2.2
3	/ʏ-ɪ/	(69.4%)	SE 1.5	/i-y/	(73.4%)	SE 1.7
	/a-ɑ/	(69.8%)	SE 2.1	/ʏ-y/	(73.8%)	SE 2.0
4	/i-y/	(78.4%)	SE 2.2			

## Discussion

We examined whether the size and/or acoustic properties of native vowel inventories relative to the target language aid or impede L2 perceptual difficulties by directly comparing L2 vowel discrimination and categorization patterns by two listener groups with varying vowel inventory sizes. The study also tested whether cross-linguistic LDA and stepwise models were predictive of AusE and PS listeners' vowel classification patterns, which in turn should predict their discrimination patterns. Based on a larger and more complex native vowel inventory, AusE listeners were predicted to perform better overall than PS listeners due to their vowel inventory being larger than PS. While there was no effect of language background, an interaction of language background and contrast was observed, with results suggesting that vowel inventory size does not fully explain non-native vowel discrimination. In fact, PS listeners were marginally better than AusE listeners in their discrimination of Dutch /ɪ-i/, while there was a trend for AusE listeners having a higher accuracy in only one contrast, namely /ʏ-ɪ/.

Our findings are in line with our acoustic predictions that further support L2LP's tenet that L1–L2 acoustic proximities predict listeners' initial perception and discrimination patterns. That is, both listener groups appear to employ perceptual cues from their L1 when perceiving non-native sounds. Specifically, AusE and PS listeners' perceptual patterns were influenced by L1–L2 acoustic differences and both listener groups faced comparable difficulty in their perception of non-native Dutch vowels. As predicted by our cross-language LDA models, AusE listeners found Dutch /ʏ-y/ their second most challenging contrast. AusE listeners mapped this contrast across multiple native categories, Dutch /ʏ/ as AusE /ε-ʊ-ʉː/ and Dutch /y/ as AusE /ʉː-ʊ-ɪ/, leading to an overall lower discrimination performance compared to PS listeners who mapped these vowels across across two acoustically distinct native categories, PS /e/ and /i/, respectively. Further, evidence for acoustic L1–L2 overlap across multiple native categories affecting listeners' perceptual patterns, irrelevant of their L1 vowel inventory size, can be observed for Dutch /ɪ-i/. Even though AusE has two and PS one /i/ vowel, both listener groups had difficulty when discriminating this contrast. As predicted by the stepwise DAs, PS listeners employed F2 and F3 to classify Dutch /ɪ/ across two native categories, namely /i/ and /e/ while Dutch /i/ was classified solely as PS /i/. On the other hand, AusE listeners mapped Dutch /ɪ-i/ predominantly as AusE /ɪ/ but also /ε/, /i/, /ʉ:/, and /ʊ/.

These results are in line with those of earlier studies that show English listeners' initial perceptual patterns are primarily influenced by spectral cues when perceiving the Dutch tense-lax /i-ɪ/ contrast, providing further evidence that acoustic properties influence listeners' perceptual patterns of non-native sounds (e.g., Lengeris, 2008). Research has established that in their perception of high-front vowels listeners are more sensitive to vowel-intrinsic formant movement than duration (e.g., Tiffany, 1953; Stevens and House, 1963; Bennett, 1968). Specifically, English listeners are almost entirely unaffected by changes in duration for vowel contrasts such as /i-ɪ/, /e-ɪ-ε/, and /u-ʊ/, even though a large and noticeable difference exists in the production of these vowels (Hillenbrand and Nearey, 1999). Thus, perceptual evidence suggests that even though “vowel duration varies substantially across individual vowel categories the degree to which a given vowel can be distinguished from its neighbors is based on spectral characteristics” (Hillenbrand, 2013, p.25). AusE listeners' low discrimination performance and categorization of Dutch /ɪ-i/ across multiple native categories was therefore due to a higher acoustic overlap between the AusE and Dutch categories, compared to PS. As a result, PS listeners outperformed AusE listeners in their discrimination of Dutch /ɪ-i/ who found this their most challenging contrast to discern.

AusE listeners' perceptual patterns are also reflective of acoustic overlap between the number of referents available to AusE listeners. In the present study, AusE listeners were given more response categories compared to PS listeners as AusE has a larger vowel inventory. Earlier studies have shown that vowel categorization is affected by the number of mental representations available to a listener (e.g., Benders et al., 2012; Elvin et al., 2014). For instance, PS listeners were less accurate in their categorization of Spanish /i-e/ when given more response categories, /a-e-i-o-u/, compared to fewer response categories, /i/ and /e/ (Benders et al., 2012). Listeners who were given two response categories were found to be more sensitive to F1 changes allowing for an early boundary shift, while those with five options were found to constrain their sensitivity to acoustic context effects resulting in a slower boundary shift. Specifically, the authors argue that listeners who were given the response option /a/ activated more mental representations and were implicitly expecting to hear /a/, thus delaying the boundary shift between /i/ and /e/. Further, evidence of the number of mental representations and acoustic overlap affecting perceptual performance is suggested by Elvin et al. (2014). The authors suggest that AusE listeners' overall lower accuracy of BP vowels may be due to the possibility that a larger number of mental representations are activated for AusE than IS listeners. Moreover, AusE listeners' discrimination accuracy was lower for the non-native vowel contrasts that bear complete or partial acoustic overlap to native categories. As a result, AusE listeners mapped non-native sounds across two or more native categories (e.g., e-i and /o-u/). However, listeners' accuracy was not affected for contrasts that were mapped across multiple native categories but involved no acoustic neutralization (e.g., /a-ε/).

Our results further support findings by Elvin et al. (2014) as AusE listeners showed higher discrimination accuracy for contrasts involving Subset Scenario, in which listeners equate a non-native sound across two or more native categories, with minimal to no acoustic overlap. As predicted, AusE listeners had low discrimination difficulty for Dutch /i-y/ and found this their least challenging contrast. Based on the LDA model predictions, listeners were predicted to classify this contrast across two native categories, namely /ɪ-ʉː/. Non-native vowel categorization results show that AusE listeners appear to utilize all acoustically close native vowel categories in their perceptual differentiation of Dutch /i-y/. That is, listeners categorized Dutch /i/ as AusE /ɪ/, /i/, and /ε/, while /y/ was categorized as AusE /ʉː/, /ʊ/, and /ɪ/. As presented in Table 3, while there was some acoustic overlap to AusE /ʉ:/ based on F1, listeners appear to exploit backness (F2) and rounding (F3) differences to distinguish this contrast. Similarly, PS listeners' high discrimination accuracy shows that listeners also exploit F2 and F3 differences in their perception of Dutch /i-y/. That is, PS listeners categorized Dutch /i/ to PS /i/ and Dutch /y/ as PS /i/ and /u/. These results indicate that while there is acoustic overlap between Dutch /i/ and PS /i/ across all three acoustic dimensions, F2, and F3 appear to be the defining cues for PS listeners' perception of Dutch /y/.

It is well-known that PS listeners face New Scenario in their perception of Dutch /a-ɑ/ and equate this contrast as PS /a/, resulting in low discrimination accuracy (e.g., Escudero and Williams, 2012). While PS listeners did face New Scenario for this contrast, AusE listeners encountered Subset Scenario with low perceptual overlap, leading to an overall higher accuracy percentage compared to PS listeners. Our findings are in line with L2LP which stipulates that Subset Scenario should be difficult for non-native listeners, but less difficult than the New Scenario. A similar pattern can be observed for Dutch /ʏ-ɪ/. AusE listeners faced Subset Scenario by perceptually mapping Dutch /ʏ-ɪ/ across multiple native categories. However, as predicted by the stepwise classifications, AusE listeners made use of F2 and F3 differences between Dutch /ʏ-ɪ/ to discern the contrast. Conversely, PS listeners were predicted to have lower discrimination accuracy for this contrast as both Dutch vowels exhibit acoustic overlap across all three acoustic dimensions. Our predictions were borne out, as listeners found this one of their most challenging contrasts to discern facing Subset Scenario by predominantly mapping Dutch /ʏ-ɪ/ as PS /i, e, u/ leading to a lower mean accuracy compared to AusE listeners. Furthermore, AusE listeners mapped Dutch /ʏ-y/ across multiple native categories, namely /ε/, /ɪ/, /ʊ/, and /ʉ:/. Due to an acoustic overlap across all three acoustic cues, AusE listeners found this their second most challenging contrast. PS listeners categorized Dutch /ʏ-y/ predominantly to native /e/ and /i/, encountering Similar Scenario.

In line with L2LP, our perceptual results suggest that for both listener groups, non-native phonemes are easier to discern when they are in acoustic proximity to distinct native categories and are categorized across acoustically similar native counterparts. In addition, listeners with larger vowel inventories seem to activate multiple native categories reflected in the perceptual patterns of some L2 vowels. This demonstrates that for the most part, having a larger and more complex first-language vowel inventory is not a good predictor for L2 perceptual difficulties as reported in previous literature (e.g., Iverson and Evans, 2007, 2009). Furthermore, activation of multiple native categories for non-native contrasts involving acoustic or perceptual overlap results in lower discrimination accuracy, such as categorization of Dutch /a-ɑ/ and /ʏ-ɪ/ for PS listeners, while medium to good discrimination is seen for contrasts that are not completely neutralized across native categories, such as categorization of Dutch /i-y/, /a-ɑ/, and /ʏ-ɪ/ for AusE listeners. Findings further suggest that both listener groups transfer perceptual cues from their native language when discriminating non-native contrasts. Moreover, rather than overall LDA classifications, our findings suggest that individual cues offer a more detailed insight into naïve listeners' perceptual patterns. That is, as established in earlier studies, listeners with varying L1 vowel inventories appear to access the complex interaction of spectral and temporal information in their perception of L2 sounds (Lengeris, 2008). Thus, our findings are in line with L2LP model's acoustic hypothesis that stipulates that L1–L2 acoustic relationships are predictive of listeners' initial perceptual patterns, as well as previous research that demonstrates acoustic proximities rather than vowel inventory size offering more detailed non-native/L2 perceptual pattern predictions (e.g., Escudero and Williams, 2011; Elvin et al., 2014; Escudero et al., 2014).

Nevertheless, further analyses should be undertaken to generate more accurate perceptual predictions based on quantitative measures of cross-linguistic similarity between the target language and listeners' L1, such as Euclidean Distances. While the present study uses F1, F2, and F3 measurements reported in earlier studies, vowel-extrinsic speaker normalization procedures (e.g., Lobanov, 1971) require fundamental frequency (F0) in addition to the first three formant measurement as a means of computing average formant values across speakers. Since F0 values were not available in the AusE corpora used in the present study, we were unable to compute formant means across genders as F0-values, which are part of a detailed ED comparison between L1/L2 languages. This will allow for a more detailed analysis of the present data and comparison of acoustic overlap for contrasts that exhibit Subset to that of earlier research (e.g., Elvin et al., 2014). In line with the present findings, and as suggested by one of our reviewers, an interesting avenue for future research may be to also include perception tasks that simply require listeners to write down the perceived non-native sound instead of categorizing it to a native category option.

In sum, our findings demonstrate that regardless of AusE and PS listeners' varying native vowel inventories it is the L1–L2 acoustic relationships that predict their non-native vowel perception. The findings also show that cross-linguistic LDA and stepwise models were predictive of AusE and PS listeners' vowel classification patterns, which in turn predicted their discrimination patterns. Ongoing research will further examine whether our results extend to L2 word recognition abilities in words that differ in the same Dutch vowel contrasts. Findings may inform possible future language learning programs which could include customizing individual L2 learning according to native language.

## Ethics statement

Participants were informed of the consent process and were asked to sign a consent sheet prior to commencing of the experiment. They were also advised that all participation was voluntary and that they could stop/withdraw from the study at any time without this affecting their relationship with the researchers, Western Sydney University or The MARCS Institute.

## Author contributions

The full author contribution criteria have been met by all three authors of this submission. Substantial contributions to the conception or design of the work; or the acquisition, analysis, or interpretation of data for the work; (SA, KM, PE); Drafting the work or revising it critically for important intellectual content; (SA, KM, PE); Final approval of the version to be published; (SA, KM, PE); Agreement to be accountable for all aspects of the work in ensuring that questions related to the accuracy or integrity of any part of the work are appropriately investigated and resolved (SA, KM, PE).

## Funding

This research was supported by MARCS Institute start-up funds (PE), Australian Research Council grants DP130102181 (CI, PE), and ARC Centre of Excellence for the Dynamics of Language project (CI, PE), CE140100041 (SA, KM).

### Conflict of interest statement

The authors declare that the research was conducted in the absence of any commercial or financial relationships that could be construed as a potential conflict of interest.

## Footnotes

^1^PAM and PAM-L2 describe these patterns as Single Category assimilation, Two Category assimilation, and Uncategorized assimilation, respectively (Best, 1995; Best and Tyler, 2007).

^2^The mean age for these participants was not reported by the previous authors.
